# Axin phosphorylation in both Wnt-off and Wnt-on states requires the tumor suppressor APC

**DOI:** 10.1371/journal.pgen.1007178

**Published:** 2018-02-06

**Authors:** Ofelia Tacchelly-Benites, Zhenghan Wang, Eungi Yang, Hassina Benchabane, Ai Tian, Michael P. Randall, Yashi Ahmed

**Affiliations:** Department of Molecular and Systems Biology and the Norris Cotton Cancer Center, Geisel School of Medicine at Dartmouth College, Hanover, NH, United States of America; St. Jude Children's Research Hospital, UNITED STATES

## Abstract

The aberrant activation of Wnt signal transduction initiates the development of 90% of colorectal cancers, the majority of which arise from inactivation of the tumor suppressor Adenomatous polyposis coli (APC). In the classical model for Wnt signaling, the primary role of APC is to act, together with the concentration-limiting scaffold protein Axin, in a “destruction complex” that directs the phosphorylation and consequent proteasomal degradation of the transcriptional activator β-catenin, thereby preventing signaling in the Wnt-off state. Following Wnt stimulation, Axin is recruited to a multiprotein “signalosome” required for pathway activation. Whereas it is well-documented that APC is essential in the destruction complex, APC’s role in this complex remains elusive. Here, we demonstrate in Drosophila that Axin exists in two distinct phosphorylation states in Wnt-off and Wnt-on conditions, respectively, that underlie its roles in the destruction complex and signalosome. These two Axin phosphorylation states are catalyzed by glycogen synthase kinase 3 (GSK3), and unexpectedly, completely dependent on APC in both unstimulated and Wnt-stimulated conditions. In a major revision of the classical model, we show that APC is essential not only in the destruction complex, but also for the rapid transition in Axin that occurs after Wnt stimulation and Axin’s subsequent association with the Wnt co-receptor LRP6/Arrow, one of the earliest steps in pathway activation. We propose that this novel requirement for APC in Axin regulation through phosphorylation both prevents signaling in the Wnt-off state and promotes signaling immediately following Wnt stimulation.

## Author summary

The Wnt signal transduction pathway directs fundamental cellular processes during development and in homeostasis. Wnt signaling is deregulated in 90% of colorectal cancers, most of which are triggered by inactivation of the tumor suppressor Adenomatous polyposis coli (APC). In the classical model, APC’s sole role in Wnt signaling is to target the transcriptional coactivator β-catenin for phosphorylation and subsequent degradation, and thereby to inhibit signaling in the unstimulated state. However, the mechanisms by which APC functions remain unknown. Herein, we provide evidence in Drosophila that supports a major role for APC in the direct regulation of the scaffold protein Axin in both Wnt-on and Wnt-off conditions. Our results indicate that APC promotes Axin phosphorylation, which is required not only to inhibit signaling in the unstimulated state, but also to activate signaling following Wnt stimulation. These unanticipated findings support a more active and multifaceted role for APC in Wnt signaling than previously known, and force revision of the current model for APC function.

## Introduction

The Wnt/β-catenin signal transduction pathway orchestrates fundamental cellular processes during development and in adult homeostasis [1–4]. Wnt signaling is aberrantly activated in many human cancers, including nearly all colorectal cancers, most of which are triggered by inactivation of the tumor suppressor Adenomatous polyposis coli (APC) [2,3]. In the Wnt-off state, the transcriptional activator β-catenin (Armadillo in Drosophila) is targeted for proteasomal degradation by a multiprotein “destruction complex” that includes APC, the scaffold protein Axin, and the kinases glycogen synthase kinase 3 (GSK3) and casein kinase 1α (CK1α) [5–8]. The binding of Wnt ligands to their transmembrane co-receptors, Frizzled (Fz) and low-density lipoprotein receptor protein 5/6 (herein LRP6, Arrow in Drosophila), induces both rapid phosphorylation of the cytoplasmic tail of LRP6 [9–11] and the dissociation of Axin from the destruction complex [12–19]. Specifically, LRP6 phosphorylation creates binding sites for Axin, promoting its rapid recruitment and the assembly of a membrane-associated multiprotein “signalosome” that includes GSK3 and the cytoplasmic protein Dishevelled (Dsh) [9–11,16,20–22]. Signalosome formation inhibits destruction complex activity, leading to β-catenin stabilization, nuclear translocation, and the consequent transcriptional regulation of Wnt target genes [23–25].

Axin is a concentration-limiting scaffold that promotes assembly of both the destruction complex in the Wnt-off state and the signalosome following Wnt stimulation [11,19,26–29]. Axin is phosphorylated under basal conditions and dephosphorylated after Wnt stimulation [12,18,19,30,31]. Both GSK3 and CK1 catalyze Axin phosphorylation [27,30–32]. GSK3-mediated phosphorylation of Axin enhances its interaction with other components of the destruction complex in the Wnt-off state [19,27,30] and promotes Axin’s rapid association with phospho-LRP6 following Wnt stimulation [19]. This association of phospho-Axin with phospho-LRP6 triggers Axin dephosphorylation, inducing a conformational change that inhibits Axin’s interaction with both the destruction complex and the signalosome [12,19,33]. Several hours after Wnt exposure, Axin is degraded [13,20,23,31,34–37]. In addition to phosphorylation, ADP-ribosylation catalyzed by the enzyme Tankyrase (Tnks) also regulates Axin stability and activity. Tnks-mediated ADP-ribosylation targets Axin for ubiquitin-dependent proteasomal degradation in the Wnt-off state [38] and promotes pathway activation following Wnt exposure [37]. ADP-ribosylated Axin accumulates rapidly in response to Wnt stimulation, facilitating the interaction between Axin and phospho-LRP6/Arrow [37].

In the classical model, the major role of APC is to promote the phosphorylation and consequent proteolysis of β-catenin, thereby inhibiting signaling in the Wnt-off state. Whereas APC’s essential role in destruction complex activity is well documented, the mechanism by which APC promotes this activity remains unclear. APC was initially thought to act as a scaffold for the destruction complex, but after further assessment of protein interactions within the complex, this scaffold role was attributed to Axin [5,27,39–41]. Subsequently, APC was thought to promote destruction complex activity by preventing β-catenin dephosphorylation [42]; however, the level of β-catenin phosphorylation remains high in multiple different colon cancer cell lines in which *APC* is inactivated by mutation [43]. Furthermore, loss-of-function studies in Drosophila demonstrated that APC is required not only to inhibit signaling in the Wnt-off state but also to promote signaling following Wnt stimulation in multiple different tissues, suggesting that the roles of APC are broader than proposed in the classical model [44].

Herein, we present three lines of evidence in Drosophila that support a major role for APC in the regulation of Axin in both Wnt-on and Wnt-off conditions. First, we identify two distinct states of Axin phosphorylation in unstimulated and Wnt-stimulated conditions, respectively: Axin is “fully” phosphorylated by GSK3 in the Wnt-off state and partially phosphorylated following Wnt stimulation. Second, we demonstrate that APC is essential for this GSK3-catalyzed phosphorylation of Axin in both the Wnt-off and Wnt-on states. Third, we find that following Wnt stimulation, the rapid transition in Axin activity and its association with activated phospho-LRP6/Arrow are dependent on APC. Together, these findings reveal a novel role for APC in Axin regulation by phosphorylation that not only prevents signaling in the Wnt-off state, but also activates signaling following Wnt stimulation.

## Results

### *In vivo* analysis of domains required for the rapid regulation of Axin following Wingless exposure

We sought to identify the mechanisms that rapidly regulate Axin during the transition in its roles from the destruction complex to the signalosome after Wnt exposure. We utilized Drosophila embryos, in which Wingless (Wg) is expressed in 14 segmental ectodermal stripes at three hours of development, providing an *in vivo* model for studying the immediate signaling events triggered by Wingless exposure [45,46]. The initial response of Axin to Wingless exposure is evident in a previously described *in vivo* system in which a maternal *α-tubulin* enhancer (*mat-Gal4*) [47] expresses Axin-V5 within two-fold of endogenous Axin levels [37]. This analysis revealed that the previously known degradation of Axin that occurs in Wingless/Wnt-responding cells several hours after stimulation [13,20,23,31,34–37] is preceded by an earlier phase that occurs within 30 minutes of Wingless exposure, during which Axin accumulates rapidly in Wingless-responding cells, which also have increased levels of Armadillo/β-catenin [37]. Evidence supporting an increase in endogenous Axin levels following Wingless stimulation was provided by biochemical analyses of lysates from embryos collected at stages that immediately precede or follow the onset of Wingless expression [37].

To identify the domains required for Axin regulation in response to Wingless stimulation, we performed a structure-function analysis in this *in vivo* model. We generated *Axin* transgenes with deletions in domains required for Axin’s interaction with other Wingless pathway components: the ADP-ribose polymerase Tankyrase (*AxinΔTBD-V5*), the tumor suppressor Apc (*AxinΔRGS-V5*), the transcriptional activator Armadillo (*AxinΔARM-V5*), the phosphatase PP2 (*AxinΔPP2-V5*), and the signalosome component Dishevelled (*AxinΔDIX-V5*) (S1 Table). These *Axin* transgenes were integrated at the same genomic site to permit their direct comparison in the absence of transcriptional position effects.

**Fig 1:**
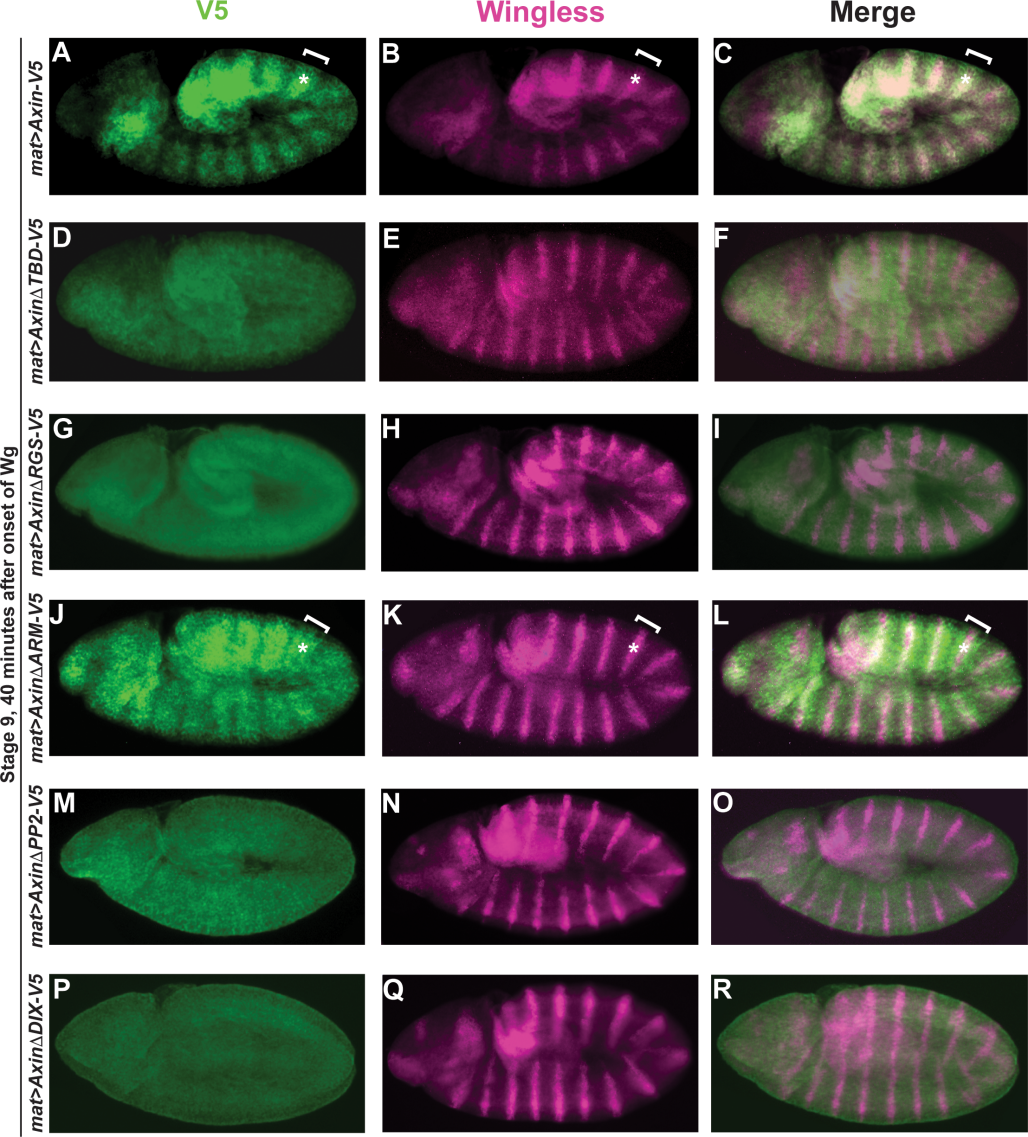
Functional analysis of Axin domains required for its stabilization following Wingless stimulation. Confocal images of stage 9 embryos expressing indicated transgene with the *mat-Gal4* driver. Genotypes at left margin, antibodies on top. (A-C) By 40 minutes after the onset of Wg expression in segmental stripes, Axin-V5 is distributed in wide segmental stripes (brackets) that overlap with the Wg stripes (asterisks), indicating that Axin levels increase rapidly in cells responding to Wg exposure. (D-R) AxinΔTBD-V5 (D-F), AxinΔRGS-V5 (G-I), AxinΔPP2-V5 (M-O) and AxinΔDIX-V5 (P-R) are uniformly increased throughout the embryonic ectoderm; in contrast, AxinΔArm-V5 (J-L) is distributed in wide segmental stripes (brackets) that overlap the Wg stripes (asterisks). The Tankyrase-binding domain (TBD), Apc-binding domain (RGS), PP2A-binding domain (PP2), and Dishevelled-binding domain (DIX) are required for the initial accumulation of Axin in Wg-responding cells, whereas the Arm-binding domain (ARM) is dispensable for this process. Due to variation in staining intensity between embryos, the relative level of Axin in the stripes and interstripes can be assessed within a single embryo, but not between different embryos. For all images, anterior left, dorsal up.

Next, we examined the effects of the Axin deletions during both the initial stage, when Axin accumulates rapidly in Wingless-responding cells (Stage 9, 40 minutes after the onset of Wingless expression in segmental stripes), and two hours later, when Axin is degraded in the same cells (Stage 10, 120 minutes after the onset of Wingless). As reported previously [37], full-length Axin-V5 was distributed uniformly throughout the ectoderm prior to Wingless expression (S2A–S2C Fig); however, by 30 minutes after Wingless exposure, Axin-V5 accumulated in wide segmental stripes in cells responding to Wingless stimulation (Fig 1A–1C). In contrast, by 120 minutes after Wingless exposure, Axin was degraded in these Wingless-responding cells, as described previously (S1D–S1F Fig) [34,37]. Consistent with previous findings [37], deletion of the Tankyrase binding domain in Axin (*AxinΔTBD-V5*) resulted in aberrant Axin stabilization in all ectodermal cells and loss of Axin’s initial accumulation in stripes (Fig 1D–1F), whereas the subsequent Wingless-dependent proteolysis of Axin occurred normally (compare S2A–S2C Fig and S1D–S1F Fig). Similarly, we found that deletion of the Apc binding domain (*AxinΔRGS-V5*), the PP2 binding domain (*AxinΔPP2-V5*), and the Dishevelled binding domain (*AxinΔDIX-V5*) each resulted in loss of the early accumulation of Axin in Wingless-responding cells (Fig 1G–1I, 1M–1O and 1P–1R respectively and S1 Table), but did not inhibit the subsequent Wingless-dependent Axin degradation (S2D–S2F, S2J–S2L, S2M–S2O Fig respectively). In contrast, the Armadillo binding domain (*AxinΔARM-V5*) was neither required for the initial accumulation of Axin in segmental stripes nor for its subsequent Wingless-dependent degradation (Fig 1J–1L, S2G–S2I Fig and S1 Table). Taken together, these results indicate that the TBD, RGS, PP2 and DIX domains are necessary, whereas the Armadillo binding domain is dispensable, for the rapid accumulation of Axin following Wingless stimulation *in vivo*.

**Fig 2:**
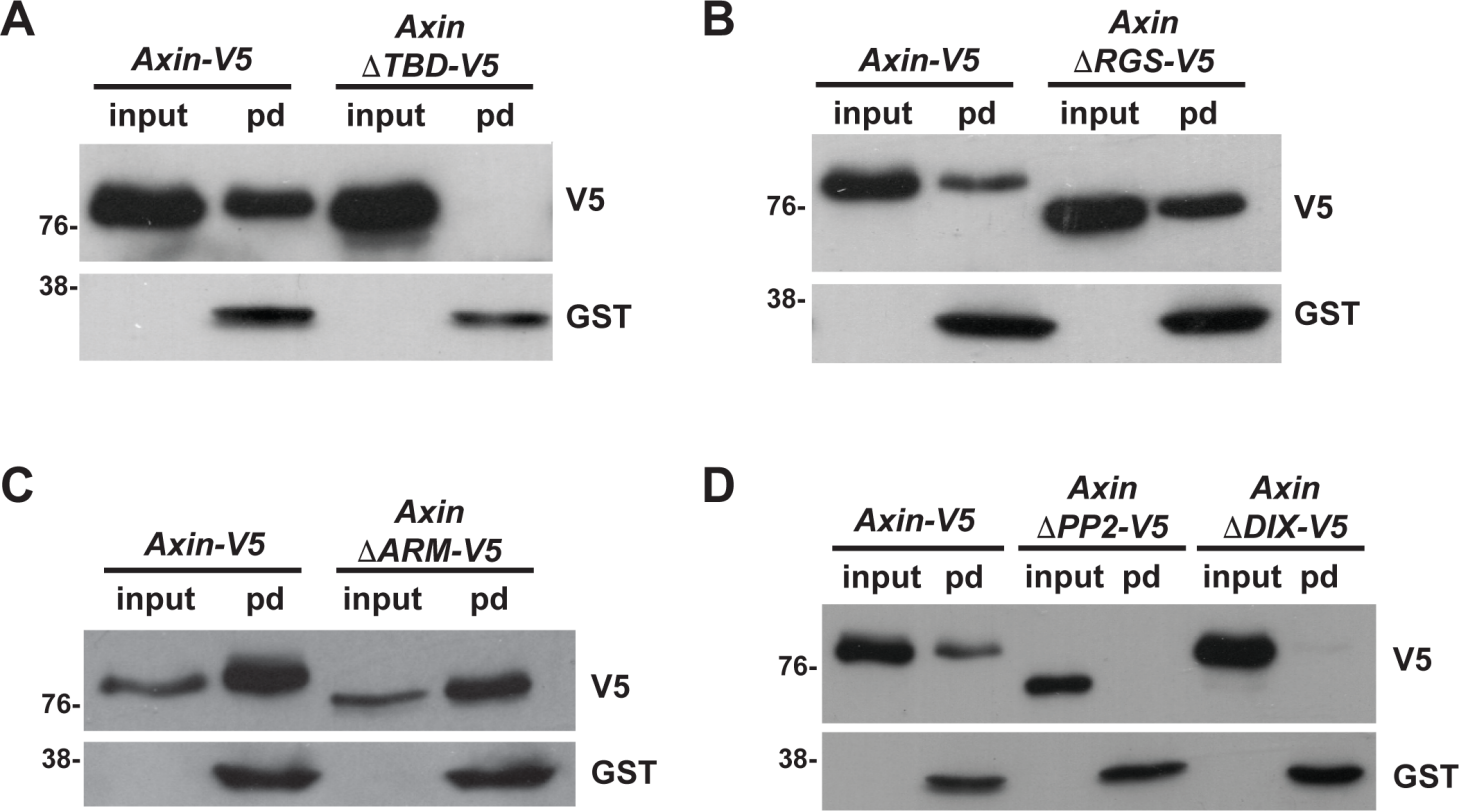
The Apc binding domain is required for the rapid regulation of Axin in Wingless-responding cells, but dispensable for Axin ADP-ribosylation. Use of the WWE pull-down assay to identify Axin domains that are important for ADP-ribosylation. Lysates from third instar larvae expressing indicated transgene with the *C765-Gal4* driver were incubated with GST-WWE beads. Axin-V5 is pulled down (pd) by WWE (A), indicating that Axin-V5 is ADP-ribosylated. AxinΔTBD-V5 (A), AxinΔPP2-V5 and AxinΔDIX-V5 (D) are not pulled down by WWE, whereas AxinΔRGS-V5 (B) and AxinΔArm-V5 (C) are pulled down by WWE, indicating that the Tnks, PP2A and Dsh binding domains are required for Axin ADP-ribosylation, whereas the Apc and Armadillo binding domains are dispensable.

### ADP-ribosylation is necessary but not sufficient for the rapid regulation of Axin following Wingless exposure

As Tnks-catalyzed ADP-ribosylation promotes the rapid accumulation of Axin in stripes of embryonic cells responding to Wingless exposure [37], we hypothesized that the loss of Axin stripes in some Axin deletion mutants resulted from decreased ADP-ribosylation. To test this hypothesis *in vivo*, we examined Axin ADP-ribosylation in lysates from larvae expressing either full length *Axin-V5* or the *Axin-V5* deletions. To detect ADP-ribosylated Axin, which is present at low levels *in vivo*, we performed pulldowns with the Trp-Trp-Glu (WWE) domain from the E3 ubiquitin ligase RNF146/Iduna, which targets Tnks substrates for proteasomal degradation [48,49]. The WWE domain of RNF146 interacts directly with the poly-ADP in Tnks substrates, and thus permits the sensitive detection of ADP-ribosylated Axin in GST-WWE pull downs (pd) [49,50]. Previous work verified the specificity of this assay, as the WWE pull down of ADP-ribosylated Axin is abrogated in *Tnks* null mutants [51]. As expected, ADP-ribosylated Axin was readily detected in GST-WWE pulldowns from lysates of larvae expressing full-length *Axin-V5*, but not in those expressing *AxinΔTBD-V5*, in which the Tnks binding domain had been deleted ([37] and Fig 2A). Furthermore, analysis of lysates from larvae expressing *AxinΔRGS-V5* or *AxinΔARM-V5* revealed that both the Apc binding domain and the Armadillo binding domain were dispensable for Axin ADP-ribosylation (Fig 2B and 2C and S1 Table). In contrast, the PP2 and Dishevelled binding domains were required (Fig 2D and S1 Table). These findings indicate that the three Axin deletions that are not ADP-ribosylated (AxinΔTBD-V5, AxinΔPP2-V5, and AxinΔDIX-V5) do not accumulate in stripes following Wingless exposure, and instead are aberrantly stabilized in all ectodermal cells (S1 Table). In contrast, AxinΔARM-V5, which retains ADP-ribosylation, also retains the ability to rapidly accumulate in stripes in Wingless-responding cells (S1 Table). These data suggest that ADP-ribosylation is indeed required for the rapid accumulation of Axin in Wingless-responding cells. Unexpectedly however, AxinΔRGS-V5, another deletion that blocks the accumulation of Axin in Wingless-responding cells, did not inhibit ADP-ribosylation (Fig 2B, S1 Table). Together, these results provide evidence that ADP-ribosylation is required but not sufficient for the rapid regulation of Axin in cells responding to Wingless stimulation.

### Apc is essential for the rapid regulation of Axin that follows Wingless exposure

**Fig 3:**
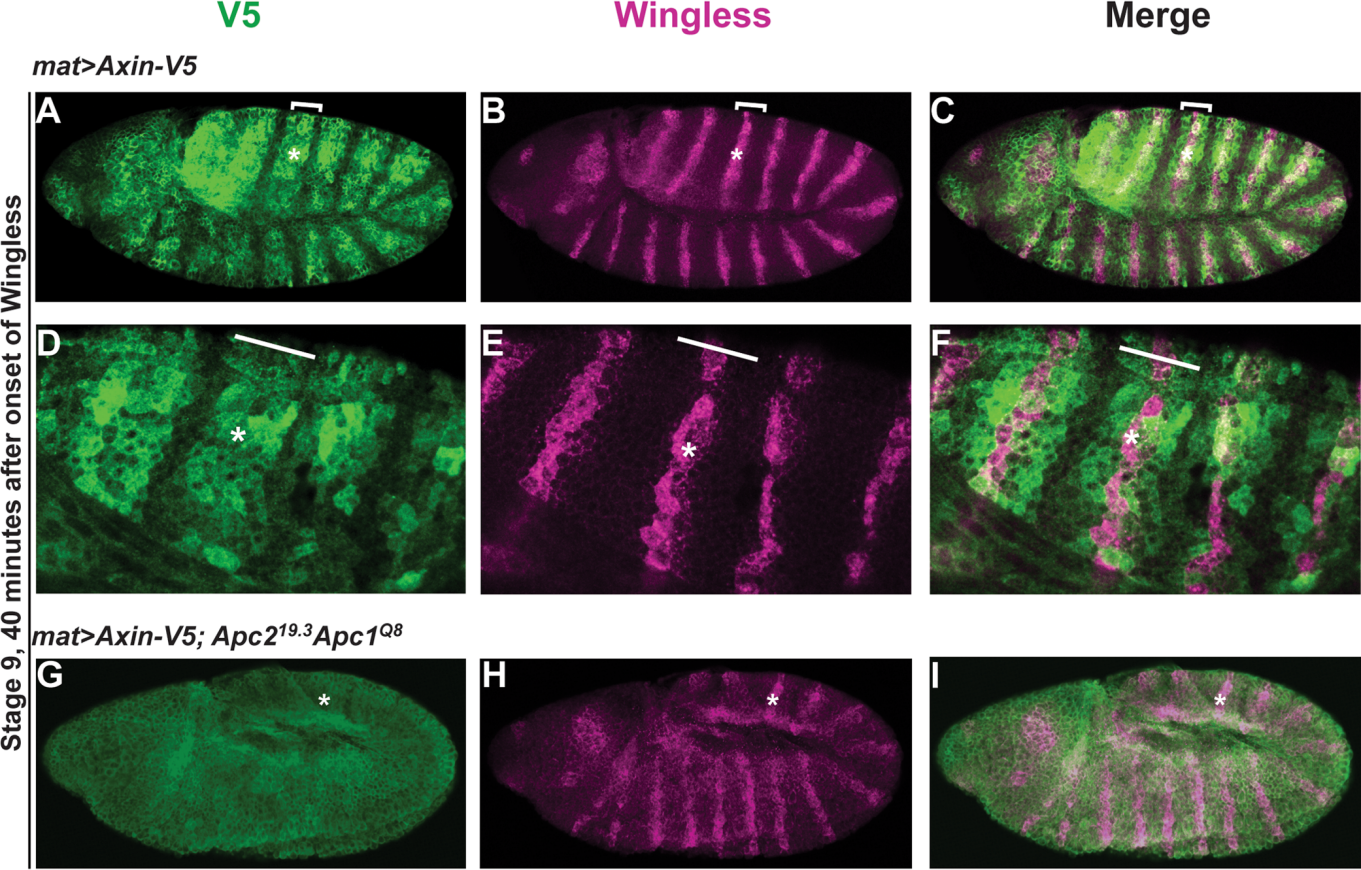
Apc promotes the rapid regulation of Axin following Wingless exposure. (A-F) Axin accumulates in Wingless-responding cells. Stage 9 embryos expressing *Axin-V5* driven by the *mat-Gal4* driver, co-immunostained with V5 and Wg antibodies. By 40 minutes after the onset of Wg exposure, Axin-V5 is distributed in wide segmental stripes (brackets) that overlap the narrow Wg stripes (asterisks). Higher magnification images are shown in (D-F). (G-I) Stage 9 embryos in which *Apc2* is inactivated both maternally and zygotically and *Apc1* is reduced zygotically. The genotype of the mother of these embryos is *mat-Gal4*/UAS-Axin-V5; *Apc2*^*19*.*3*^*/Apc2*^*19*.*3*^ and the genotype of the father is UAS-Axin-V5/UAS-Axin-V5; *Apc2*^*19*.*3*^ *Apc1*^*Q8*^*/TM6B*. In contrast with wild-type, Axin-V5 is uniformly increased in all ectodermal cells, indicating Apc is required for the initial accumulation of Axin in Wingless-responding cells.

The accumulation of Axin in stripes in Wingless-responding cells correlates temporally with its role in the activation of Wingless signaling [37]. As the Apc binding domain of Axin is essential for accumulation of Axin in these stripes (Fig 1G–1I), we hypothesized that Apc may play a novel role in the rapid regulation of Axin following Wingless exposure. To test this hypothesis, we examined the role of Apc in the formation of the Axin-V5 stripes. For that purpose, we used mutant embryos in which Apc2 is eliminated both maternally and zygotically and Apc1 is reduced zygotically (see Methods). In these *Apc* mutants, the early Axin stripes did not form and Axin was aberrantly stabilized in all ectodermal cells (Fig 3G–3I), in sharp contrast with wild-type embryos (Fig 3A–3F). These results suggest that Axin stabilization in Wingless-responding cells may result from inhibition of Apc-mediated Axin degradation. However, the subsequent Wingless-dependent proteolysis of Axin was not affected by Apc depletion (S3 Fig). Thus both Apc depletion and deletion of the Apc-binding domain of Axin prevent formation of the initial Axin stripes in Wingless-responding cells (Fig 3 and Fig 1), but have no effect on the later Wingless-dependent proteolysis of Axin (S3 Fig and S2 Fig). Together, these findings suggest that Apc, and its interaction with Axin are essential for the rapid regulation of Axin in Wingless-responding cells, but dispensable for the Axin degradation that occurs hours after Wingless stimulation.

**Fig 4:**
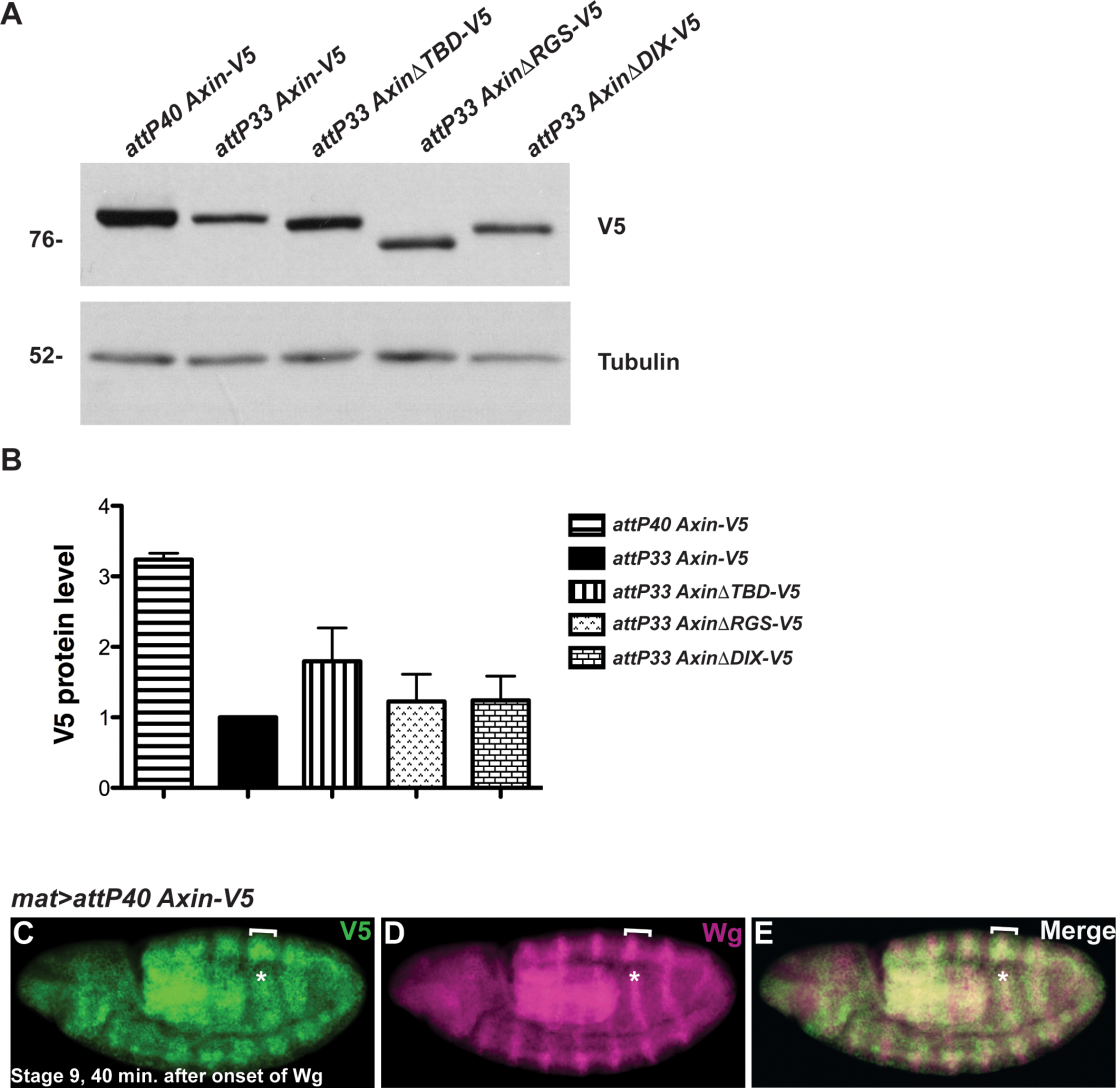
Increased basal levels of Axin do not preclude the accumulation of Axin in stripes in Wingless-responding cells. (A) Immunoblot of lysates from embryos expressing indicated transgenes. Lysates from embryos collected at 0–2 hours of development, prior to the onset of Wg expression. Integration of the *Axin-V5* transgene at *attP40* site results in higher levels than other transgenes. Tubulin was used as a loading control. (B) Quantification of the relative levels of indicated proteins expressed in embryos (0–2 hours). Results represent three independent experiments. Values indicate mean ± SD. (C-E) Immunostaining of stage 9 embryos expressing *attP40 Axin-V5* driven by the *mat-Gal4* driver with V5 and Wg antibodies. By 40 minutes after onset of Wg exposure, Axin-V5 accumulates in wide segmental strips (brackets) that overlap the narrow Wg stripes (asterisks).

Apc is known to negatively regulate the basal levels of Axin *in vivo*; in the absence of Wingless stimulation, either inactivation of Apc or deletion of the Apc binding domain of Axin stabilizes Axin [29,44,52]. Therefore, we postulated that the elevated Axin levels resulting from Apc inactivation might inhibit the rapid regulation of Axin following Wingless exposure. We therefore tested whether an increase in Axin, to levels higher than those resulting from deletion of the Apc binding domain, blocks Axin regulation in response to Wingless exposure. We utilized transgenic flies in which the *Axin-V5* transgene is integrated at a genomic site (*attP40*) that is known to result in higher expression levels than the original integration site (*attP33*) [37,53]. Indeed, immunoblotting of embryonic lysates revealed that the basal levels of Axin from *Axin-V5* integrated at the *attP40* site (*attP40 Axin-V5*) were higher than the basal Axin levels in embryos expressing either full-length *Axin-V5*, *AxinΔRGS-V5*, *AxinΔTBD-V5*, or *AxinΔDIX-V5* integrated at the *attP33* site (Fig 4A and 4B). Despite the elevated Axin levels in *attP40 Axin-V5* embryos, Axin stripes formed rapidly in Wingless-responding cells, and were indistinguishable from observations in embryos expressing *attP33 Axin-V5* (compare Fig 4C–4E and Fig 1A–1C, S4 Fig and S1D–S1F Fig) [37]. These findings suggest loss of the rapid Wingless-dependent regulation of Axin is not merely a consequence of increased basal Axin levels. Instead, these data provide additional evidence that Apc, and its interaction with Axin, are important for the rapid regulation of Axin that follows Wingless stimulation [44,52]. We speculate that the stabilization of Axin in Wingless-responding cells results from inhibition of Apc-dependent Axin degradation.

### Two distinct phosphorylation states of Axin exist in Wnt-on or Wnt-off conditions

**Fig 5:**
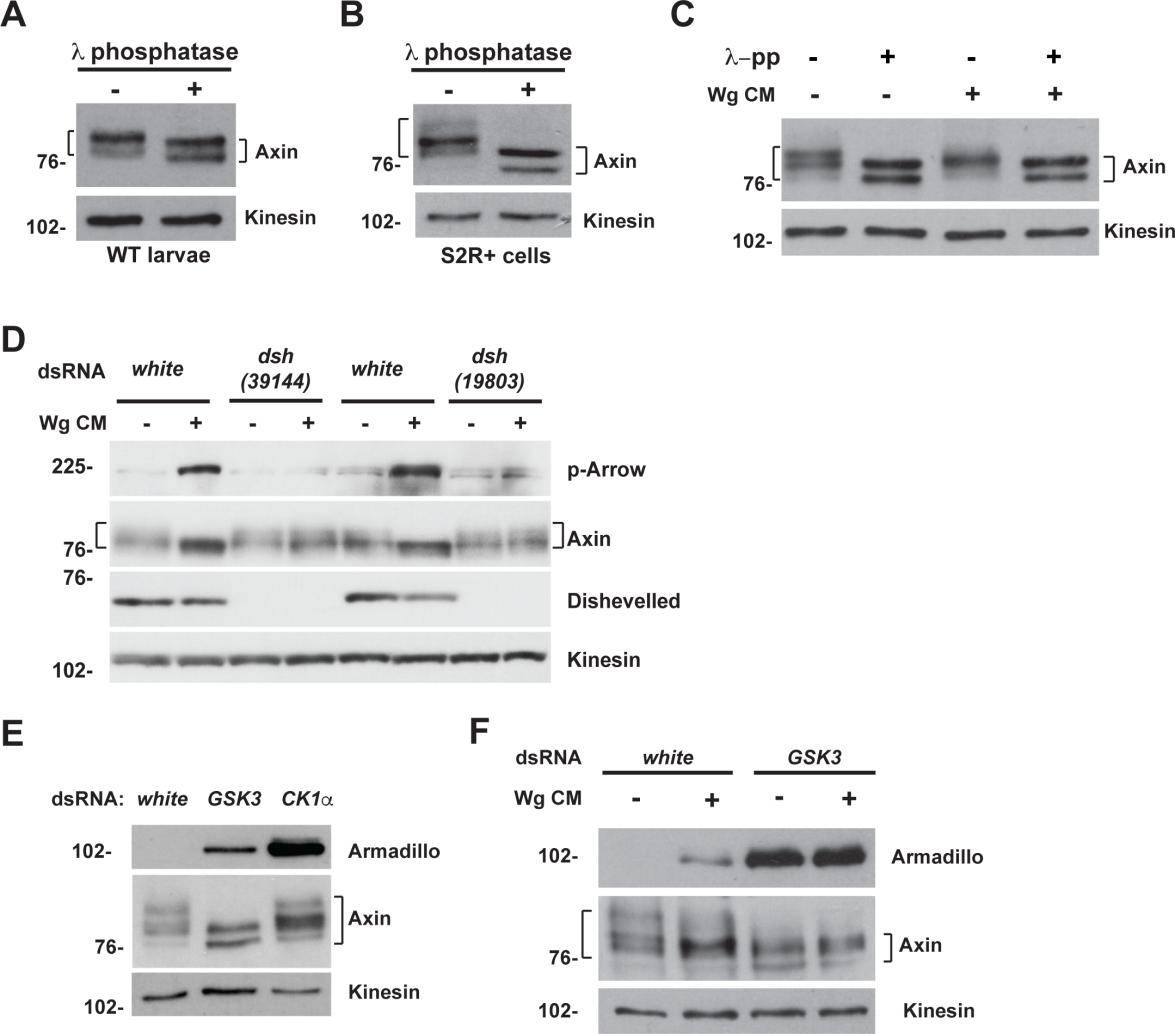
GSK3 is required for Axin phosphorylation in both unstimulated and Wingless-stimulated states. (A) Wild-type third instar larval lysates treated with λ protein phosphatase and analyzed by immunoblotting with Axin antibody. (B) S2R+ cell lysates treated with λ protein phosphatase and analyzed by immunoblotting with Axin antibody. (C) Drosophila S2R+ cells treated with Wg CM and λ protein phosphatase. Immunoblotting of cells lysates with Axin antibody revealed a downshift in Axin migration following one hour of Wingless stimulation (compare lanes 1 and 3). Phosphatase treatment results in a further downward shift in Axin mobility as compared to exposure to Wg CM (compare lanes 3 and 4). The shift in Axin migration following phosphatase treatment is the same in cells exposed to Wg CM compared to unstimulated cells (compare lanes 2 and 4). (D) RNAi-mediated knockdown of either the *white* control or *dishevelled (dsh)* in S2R+ cells reveals that the downshift of Axin in the presence of Wg CM requires activation of the Wingless pathway. (E) Immunoblot of lysates from S2R+ cells treated with indicated dsRNA. Knockdown of GSK3, but not CK1α, results in dephosphorylation of Axin detected by the Axin antibody. Armadillo/β-catenin is a positive control for effectiveness of GSK3 and CK1α knockdown. (F) Immunoblot of lysates from S2R+ cells treated with dsRNA against the *white* control or *GSK3*, followed by the treatment with control medium or Wg CM. By comparison with Wingless stimulation, GSK3 knockdown results in additional dephosphorylation of Axin, and is similar to treatment with phosphatase, indicating that nearly all phosphorylation detected by the Axin antibody is GSK3-dependent. Armadillo/β-catenin is a positive control for effectiveness of GSK3 knockdown. Kinesin was used as a loading control.

We sought to further investigate the mechanism underlying this novel role for Apc in the rapid transition of Axin in Wingless-responding cells. However, elucidation of Axin regulation under physiological conditions has been hindered by challenges in detecting the low levels of endogenous Axin. To overcome this obstacle, we capitalized on antibodies capable of detecting endogenous Drosophila Axin [54]. In lysates from both third instar larvae and Drosophila S2R+ cells, we observed several forms of Axin with distinct mobility in SDS-PAGE (Fig 5A and 5B). Axin is known to be highly regulated by phosphorylation, which is critical for its function [12,19]. To test whether the distinct forms of Axin we observed in SDS-PAGE resulted from differential phosphorylation, we treated lysates from either larvae or Drosophila S2R+ cells with lambda protein phosphatase (λ-pp). Immunoblots with Axin antibody revealed a downward shift of Axin bands after treatment with λ-pp (Fig 5A and 5B). Axin migrated as two bands after λ-pp treatment, which may reflect its known post-translational modifications other than phosphorylation [38,55–57]. These results reveal that the Axin antibody detects the presence of distinct phosphorylated forms of endogenous Axin.

Supporting previous findings that Wnt exposure induces dephosphorylation of mammalian Axin [12,31], a downward shift in the mobility of Drosophila Axin was observed following exposure of S2R+ cells to Wingless conditioned medium for one hour (Wg CM) (Fig 5C and 5D). Activation of the pathway by Wingless exposure was confirmed by the accumulation of phospho-Arrow (Fig 5D). To determine whether this shift in Axin mobility was indeed dependent on activation of the Wingless pathway, we used RNA-mediated interference to knock down Dishevelled (Dsh), an essential signalosome component [22,58–60]. dsRNA targeting of Dsh resulted in a marked decrease in its endogenous levels, and also abrogated Arrow phosphorylation following Wingless exposure (Fig 5D). Furthermore, in contrast with controls, Dsh knockdown abrogated the mobility shift in Axin following Wingless exposure (Fig 5D). To rule out off-target effects of the RNAi-mediated Dsh knockdown, we repeated this experiment with an independently derived dsRNA that targets Dsh, and again observed inhibition of the shift in Axin’s mobility following Wingless treatment (Fig 5D). To confirm that the downward shift in Axin induced by Wingless exposure resulted from its dephosphorylation, we tested the effect of phosphatase treatment. Treatment of S2R+ cell lysates with λ-pp resulted in no further shift in Axin in the presence of Wg CM, as compared to the absence of Wg CM (compare lanes 2 and 4 in Fig 5C), strongly suggesting that the Wingless-dependent mobility shift in Axin resulted from dephosphoryation. Together, these findings confirm that pathway activation is essential for Axin dephosphorylation following Wingless exposure.

In the classical model, Axin exists in a phosphorylated state under basal conditions and in an unphosphorylated state following Wnt stimulation. Our Axin antibody allowed us to test whether Wingless exposure indeed induces the complete dephosphorylation of endogenous Axin. To examine the state of Axin phosphorylation following Wingless stimulation, we treated S2R+ cells with Wg CM and subjected the cell lysates to λ-pp treatment. Shifts in Axin mobility in SDS-PAGE confirmed that Wingless stimulation induced Axin dephosphorylation, but also revealed that Axin was further dephosphorylated after treatment with λ-pp (compare lanes 3 and 4 in Fig 5C). Thus, importantly, our Axin antibodies detected both “fully” phosphorylated Axin that is present solely in the unstimulated state, and partially dephosphorylated forms of Axin that accumulate within an hour of Wnt stimulation, as revealed by shifts in migration in SDS-PAGE. Therefore, these results expand the classical model for Axin regulation, revealing that endogenous Axin is present in at least two distinct phosphoforms that are dependent on the state of pathway activation: in the unstimulated state Axin is fully phosphorylated, whereas Wingless stimulation induces the partial, rather than complete dephosphorylation of Axin.

### Wingless stimulation induces a partial, rather than complete inhibition of GSK3-mediated Axin phosphorylation

Mammalian Axin is phosphorylated by both CK1 and GSK3 [27,30–32]. To determine whether either one or both of these kinases catalyze the phosphorylation of Drosophila Axin detected by our Axin antibody, we used RNAi to knock down either GSK3 (also known as Zeste-white 3 or Shaggy in Drosophila) or CK1α in S2R+ cells. To test whether the effectiveness of GSK3 and CK1α knockdown, we examined the levels of Armadillo (Arm)/β-catenin, a known substrate of both kinases for which phosphorylation results in consequent targeting for proteasomal degradation [5,6,61,62]. We found that basal Arm levels increased upon knockdown of either GSK3 or CK1α, confirming their effective knockdown (Fig 5E). GSK3 knockdown resulted in a downward shift in the migration of all Axin phosphoforms under basal conditions, which was similar to the shifts in Axin mobility observed following treatment with λ-pp (compare Fig 5B and 5E). In contrast, despite the effective knockdown of CK1α, the migration of Axin phosphoforms in SDS-PAGE was largely unchanged (Fig 5F). However, other CK1 isoforms may contribute to the Axin phosphorylation states recognized by our antibody. These findings indicate that the Axin antibody recognizes shifts in Axin mobility resulting primarily from phosphorylation by GSK3, but not CK1α.

We sought to determine if the phosphorylation of Axin that persists following Wingless stimulation requires GSK3. To examine this, we compared Axin phosphorylation in S2R+ cells treated with Wg CM and either with or without RNAi-mediated GSK3 knockdown. Immunoblot of cell lysates with Axin antibody confirmed that Wingless stimulation resulted in a partial dephosphorylation of Axin (Fig 5F). In contrast, GSK3 knockdown resulted in the complete dephosphorylation of Axin, as revealed by further shifts in Axin mobility, which were unchanged following treatment with Wg CM (Fig 5F). These results are consistent with previous findings regarding mammalian Axin, which revealed that Wnt stimulation induces Axin dephosphorylation specifically at GSK3-catalyzed phosphosites [19,30]. These results suggest that Axin phosphorylation by GSK3 is retained at specific sites but lost at others following Wingless stimulation, although conclusive testing of this hypothesis will require the generation of Drosophila Axin antibodies directed at specific Axin phosphosites. Importantly, our findings expand the classical model for Wnt signaling, as they indicate that GSK3-catalyzed Axin phosphorylation is only partially, rather than completely inhibited following Wingless stimulation. Taken together, our findings demonstrate that our Axin antibody recognizes several phosphoforms of endogenous Axin that are dependent on both GSK3 and the state of Wingless pathway activation: “fully” phosphorylated forms of Axin are present in the Wnt-off state, whereas partially phosphorylated forms of Axin are generated during the response to Wingless stimulation.

### Apc is essential for Axin phosphorylation in both the Wnt-off and Wnt-on states

**Fig 6:**
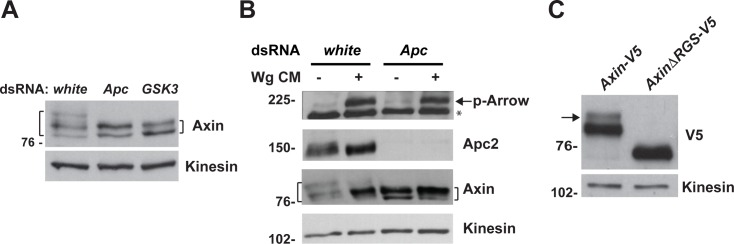
Apc promotes the phosphorylation of Axin in the absence of Wingless stimulation. (A) Immunoblot of lysates from S2R+ cells treated with dsRNA against indicated genes. Knockdown of Apc or GSK3 results in similar dephosphorylation of Axin. (B) S2R+ cells were treated with *white* or *Apc* dsRNAs, followed by the treatment with control medium or Wg CM. Immunoblotting with Axin antibody shows that Wg stimulation induces partial dephosphorylation of Axin, while knockdown of Apc results in further dephosphorylation of Axin in the absence or presence of Wg stimulation. (C) Immunoblot of lysates from third instar larvae expressing indicated transgenes with V5 antibody. Deletion of the Apc-binding domain eliminates the phosphorylated form of Axin-V5. Kinesin was used as a loading control.

As Apc is essential for the rapid regulation of Axin following Wingless stimulation *in vivo* (Fig 3), and as the regulation of Axin phosphorylation underlies Axin’s transition in response to Wnt exposure, we tested whether Apc is important for Axin phosphorylation by subjecting Drosophila S2R+ cells to RNAi-mediated depletion of Apc (Apc1 and Apc2). Unexpectedly, Apc knockdown or GSK3 knockdown had the same effect on Axin: all forms of phosphorylated Axin detected by our Axin antibody were eliminated (Fig 6A). Furthermore, treatment of Apc-depleted cells with Wg CM did not result in further dephosphorylation of Axin, consistent with the findings observed with GSK3 depletion (Fig 6B and Fig 5F). Thus, unexpectedly, these results suggest that Apc is important for GSK3-mediated Axin phosphorylation in both the Wnt-off and Wnt-on states.

To further test this conclusion, we investigated whether the Apc binding domain of Axin is required for Axin phosphorylation. We expressed either *Axin-V5* or *AxinΔRGS-V5* in wing imaginal discs of third instar larvae, and analyzed the lysates by immunoblots with V5 antibody. This analysis revealed two Axin bands of distinct mobility (Fig 6C and S5 Fig). To determine whether differential phosphorylation resulted in the differences in the mobility of these two bands, we treated larval lysates with λ-pp. Phosphatase treatment resulted in a downward shift in the mobility of the upper band, confirming that the V5 antibody detected both a phosphorylated and dephosphorylated form of Axin-V5 (S5 Fig). Furthermore, deletion of the Apc binding domain of Axin (AxinΔRGS-V5*)* resulted in loss of the phosphorylated form of Axin-V5 (Fig 6C). These results suggest that the interaction between Axin and Apc promotes Axin phosphorylation *in vivo*. Taken together with the RNAi-mediated Apc depletion experiments, these findings indicate that Apc is required for the GSK3-catalyzed phosphorylation of Axin.

### Apc promotes the interaction between Axin and phospho-LRP6/Arrow following Wingless stimulation

**Fig 7:**
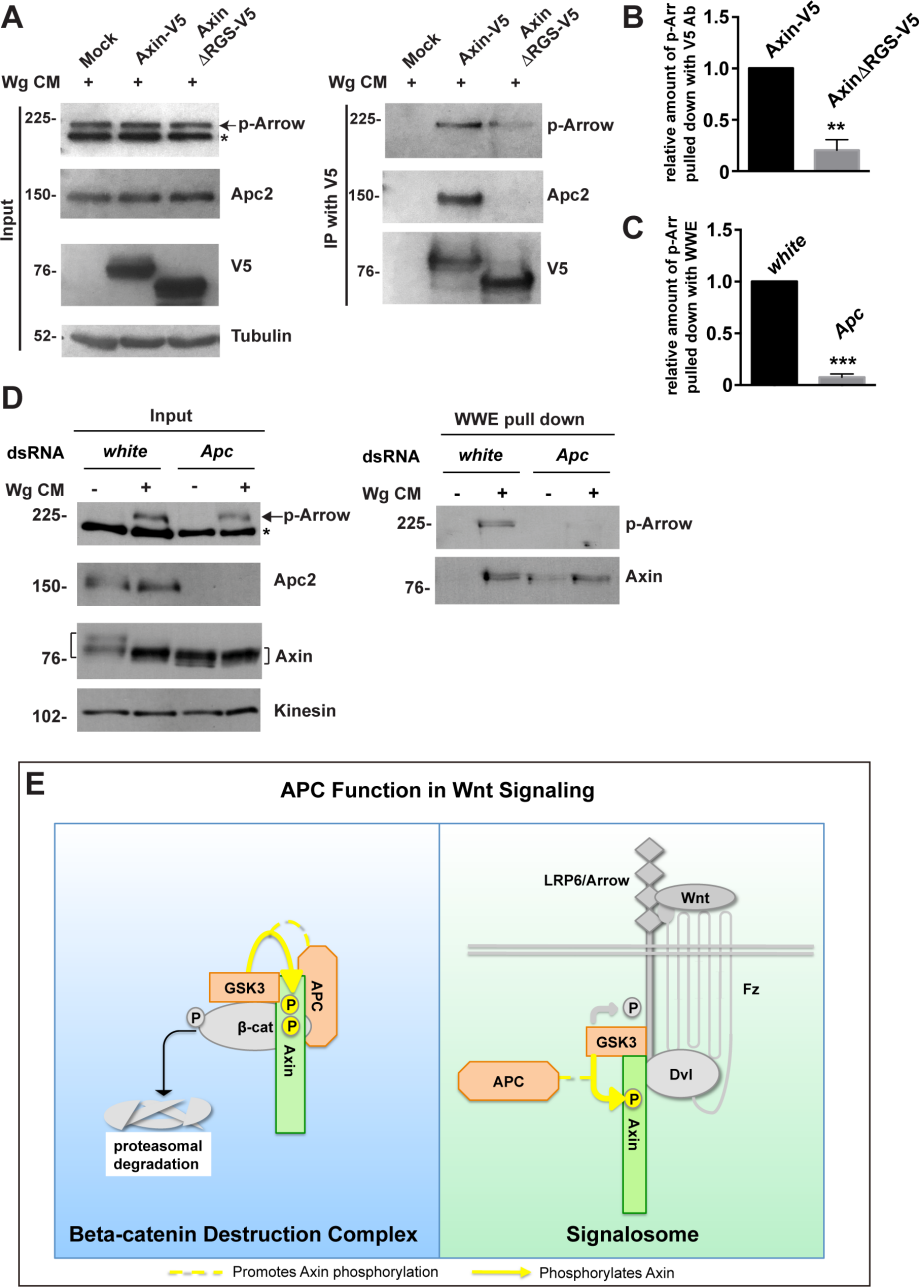
Apc promotes the association between Axin and phospho-LRP upon Wingless stimulation. (A) S2R+ cells were transfected with the indicated plasmids, and treated with Wg CM 48 hours later for one hour. Lysates were subjected to immunoprecipitation with V5 antibody and analyzed by immunoblot. Deletion of the Apc binding domain of Axin (AxinΔRGS-V5) reduced the interaction between Axin and phosphorylated LRP6/Arrow after Wingless stimulation, as revealed by immunoblot with phospho-LRP6 antibody. Tubulin was used as a loading control. (B) Quantification of relative levels of phospho-Arrow pulled down with Axin-V5 from experiment shown in (A). Error bars represent s.e.m. of three independent experiments. *P* = 0.0054. (C) Quantification of relative levels of phospho-Arrow pulled down with WWE from experiment shown in (D). Error bars represent s.e.m. of three independent experiments. *P* = 0.0004. (D) S2R+ cells were treated with the indicated dsRNAs, followed by treatment with control medium or Wg CM for one hour. Lysates were subjected to GST-WWE pull down and analyzed by immunoblot. Treatment with Wg CM markedly increases the amount of ADP-ribosylated Axin pulled down with GST-WWE. Apc knockdown abolishes the Wg-dependent increase in ADP-ribosylated Axin pulled down with GST-WWE. Upon treatment with Wg CM, phospho-Arrow is also pulled down with GST-WWE, but is significantly reduced by Apc knockdown. Kinesin was used as a loading control. (E) Working model for Apc function in Wnt signaling. Apc promotes GSK3-catalyzed Axin phosphorylation in both Wnt-off and Wnt-on states, the rapid transition in Axin following Wnt stimulation, and Axin’s subsequent association with the Wnt co-receptor LRP6/Arrow, one of the earliest steps in pathway activation. We propose that this requirement for APC in Axin regulation through phosphorylation both prevents signaling in the Wnt-off state and promotes signaling immediately following Wnt stimulation.

GSK3-catalyzed Axin phosphorylation and Tnks-catalyzed Axin ADP-ribosylation promote the interaction between Axin and phospho-LRP6 during the initial activation of Wnt signaling, and subsequently, Axin is dephosphorylated [19,37]. As our studies revealed that Apc is required for both the GSK3-mediated phosphorylation of Axin (Fig 6A) and for the rapid regulation of Axin in Wingless-responding cells (Fig 1G–1I and Fig 3G–3I), we hypothesized that Apc may therefore promote the association of Axin with phospho-LRP6/Arrow. We tested this hypothesis using two experimental approaches: co-immunoprecipitation and WWE pull down. First, we transfected Drosophila S2R+ cells with *Axin-V5* or *AxinΔRGS-V5* (in which the Apc binding domain is deleted), treated these cells with Wg CM, and subjected the cell lysates to immunoprecipitation with V5 antibody. As expected, deletion of the Apc binding domain of Axin resulted in diminished interaction between Axin and Apc (Fig 7A). Furthermore, deletion of the Apc binding domain of Axin also significantly diminished the interaction between Axin and phosphorylated Arrow following Wg exposure (Fig 7A and 7B). Of note, the interaction of Axin with LRP6/Arrow was mapped previously to regions far from the Axin RGS domain [23]. These results suggest that Apc promotes the interaction between Axin and phosphorylated Arrow.

Second, we further tested whether Apc promotes the interaction between Axin and phospho-Arrow using the WWE pull-down assay. ADP-ribosylated Axin is known to accumulate rapidly following Wnt stimulation, which facilitates the interaction between Axin and phospho-LRP6/Arrow [37]. Not only ADP-ribosylated Axin, but also phospho-Arrow is pulled down by WWE; thus the GST-WWE pull down assay permits sensitive detection of the interaction between Axin and Arrow, even at the low levels of endogenous Axin [37]. We tested whether Apc promotes the interaction between Axin and Arrow by depleting Apc using RNAi-mediated knockdown. We treated S2R+ cells with Wg CM and observed the accumulation of phospho-Arrow, indicating robust pathway activation (Fig 7D, left panel). As reported previously, WWE pull downs revealed that Wingless stimulation resulted in increased levels of ADP-ribosylated Axin, and that phospho-Arrow was simultaneously pulled down as a result of its interaction with ADP-ribosylated Axin (Fig 7D, right panel) [37]. Furthermore, by comparison with control, the interaction between ADP-ribosylated Axin and phospho-Arrow, as revealed by the amount of phospho-Arrow pulled down, was diminished significantly by depletion of Apc (Fig 7C and 7D, right panel). These results provide additional evidence supporting the hypothesis that Apc promotes the interaction between Axin and phospho-Arrow following Wingless stimulation.

Finally, this assay allowed us to also test the alternative model that Apc promotes the interaction between Axin and Arrow by enhancing Axin ADP-ribosylation. Since ADP-ribosylation promotes both the transition in Axin activity following Wingless stimulation and the interaction of Axin with phospho-Arrow [37], we examined the effect of Apc knockdown on Axin ADP-ribosylation using WWE pull downs. We found that the levels of ADP-ribosylated Axin pulled down by WWE following Wingless stimulation were unchanged by Apc knockdown (Fig 7D, right panel). Of note, neither Apc (Fig 7D) nor the Apc binding domain in Axin (Fig 2B) is essential for Axin ADP-ribosylation. Therefore Apc function has no effect on the levels of ADP-ribosylated Axin following Wingless stimulation, but is important for the robust interaction between Axin and phospho-Arrow. These findings suggest that the regulation of Axin by Apc following Wingless exposure is not mediated through ADP-ribosylation, and instead that the role of Apc in Axin phosphorylation or perhaps additional Apc-dependent mechanisms are likely critical for this process.

## Discussion

The mechanisms by which APC regulates the Wnt pathway have remained enigmatic despite intense investigation. Here, we provide evidence in a Drosophila model that APC is essential for the GSK3-catalyzed phosphorylation of Axin in both the Wnt-off and Wnt-on states. APC is also critical for the rapid reprogramming of Axin that follows Wnt exposure. These findings were enabled by three recently developed experimental approaches that have deepened our understanding of both Axin regulation and the essential role of APC in this process.

First, an *in vivo* model in Drosophila embryos has allowed unprecedented analysis of both the immediate regulation of Axin in cells responding to Wingless stimulation, as well as its subsequent regulation hours later [37]. The rapid accumulation of Axin in embryonic ectodermal cells exposed to endogenous Wingless is an *in vivo* hallmark for the initial activation of Wingless signaling as it requires Wingless activity, occurs rapidly after Wingless stimulation, and correlates with the timing of Wingless-induced Axin dephosphorylation. Using this model, we found that Apc and the Apc binding domain of Axin are essential for Axin’s rapid regulation in Wingless-responding cells, but dispensable for the subsequent Axin proteolysis that occurs hours later. These findings indicate, unexpectedly, that Apc acts not only with Axin in the destruction complex to inhibit signaling, but is also required for the rapid transition in Axin that occurs after Wingless exposure.

Second, a new antibody has enabled detection of Drosophila Axin at endogenous levels in immunoblots [54], allowing analysis of Axin regulation both before and just after Wingless exposure without the need for overexpression. With this antibody, we discovered that distinct phosphorylated forms of endogenous Axin exist in the Wnt-off and Wnt-on states, respectively. GSK3 is required for Axin phosphorylation in the Wnt-off state, in agreement with previous studies [12,31]. Furthermore, we also discovered that GSK3 is required for Axin phosphorylation that is present not only in the Wnt-off, but also in the Wnt-on state. Moreover, we discovered that the dephosphorylation of Axin that is induced following Wnt stimulation is partial rather than complete. Previous work revealed that Axin phosphorylation is retained during its initial association with LRP6 following Wnt exposure, and Axin is subsequently dephosphorylated, which prevents its interaction with both the signalosome and the destruction complex [19]. Thus the partially dephosphophorylated Axin we detected in the Wnt-on state may be the form that associates with LRP6. Importantly, we found that not only GSK3, but also Apc is essential for Axin phosphorylation in both the Wnt-off and Wnt-on states. Depletion of either GSK3 or Apc had the same effect as phosphatase treatment on Axin: both the fully and partially phosphorylated forms of Axin detected by the Axin antibody were eliminated (Fig 5B, 5C, 5E and 5F and Fig 6A and 6B). These findings suggest that APC-dependent phosphorylation of Axin is important for Axin regulation during the activation of signaling following Wingless exposure and further support previous studies demonstrating that GSK3-mediated phosphorylation of Axin is important for the association between Axin and phosphorylated LRP6 [19]. The mechanism by which APC promotes the GSK3-dependent phosphorylation of Axin requires further investigation, but may involve previously proposed functions for APC in regulating protein phosphorylation, GSK3 activity, Axin multimerization, or Axin membrane association [18,51,63–65]; alternatively, APC may prevent Axin dephosphorylation by a phosphatase such as PP1.

Third, a recently developed WWE pull-down assay [49] that isolates ADP-ribosylated Axin, which is present at low levels, has allowed detection of the association between endogenous Axin and phospho-LRP6/Arrow following Wnt stimulation [37]. Prior to the use of this assay, detection of the association of Axin with LRP6 at endogenous levels was limited to its recovery in sucrose density gradients [22,66]. As we discovered that Apc is required for the rapid transition of Axin following Wingless exposure (Fig 3), we used both WWE pull-downs and co-immunoprecipitation to test whether Apc promotes the association between Axin and phosphorylated Arrow, which is among the earliest events triggered by Wingless stimulation [20]. Both Apc depletion and deletion of the Apc binding domain in Axin significantly diminished the association between Axin and phosphorylated Arrow following Wingless stimulation (Fig 7). Previous work indicated that Wnt stimulation reduces the affinity of Axin for the destruction complex through Axin dephosphorylation [12,18,19]. However, other studies suggest that Axin and APC remain bound during signalosome formation following Wnt stimulation [3,29,36]. Our observation that APC enhances the association of Axin with Arrow/LRP6 does not discount the possibility that the APC-Axin interaction remains intact in the signalosome after Wingless stimulation; however, we have not been able to detect this interaction through co-immunoprecipitation studies. Furthermore, APC may promote the association of Axin with LRP6 through its roles in Axin phosphorylation, as described here, multimerization [64], or as yet unidentified mechanisms.

Although the requirement for APC in the destruction complex is well established, the mechanism by which APC promotes destruction complex activity remains unknown. Our results reveal a novel role for APC in promoting the GSK3-catalyzed phosphorylation of Axin in the Wnt-off state. We hypothesize that the physical interaction between APC and Axin facilitates the phosphorylation of Axin by GSK3 within the destruction complex. Previous studies indicated that APC promotes GSK3 activity [18], and that the phosphorylation of Axin promotes stability of the destruction complex [31]. Our findings are consistent with these previous results, and also indicate that APC’s role as a negative regulator of the pathway in the Wnt-off state requires its essential function in GSK3-mediated Axin phosphorylation.

Taken together, our findings have uncovered novel roles for APC in the regulation of Axin phosphorylation and in the initiation of Wingless signaling. Therefore, we propose the following three revisions to the classical model for Wnt signaling (Fig 7E). First, a major role of APC is to regulate the key scaffold protein Axin in both unstimulated and Wnt-stimulated states. Second, APC is essential for the rapid transition in Axin that occurs concomitantly with pathway activation after Wnt stimulation. Third, APC promotes the GSK3-catalyzed phosphorylation of Axin that is necessary in both Wnt-off and Wnt-on states. Therefore, our findings suggest that a key function of APC is to regulate Axin’s essential roles in both the destruction complex and the signalosome (Fig 7E). We speculate that through this novel mechanism, APC both prevents the aberrant activation of signaling in the unstimulated state and promotes physiological signaling following Wnt stimulation, supporting previous loss-of-function studies that revealed essential roles for APC in both the inhibition and the activation of Wnt signaling *in vivo* [44].

## Materials and methods

### Flies and genetics

To generate the *pUASTattB-AxinΔRGS-V5* transgene, residues T-54 through Y-168 were deleted by PCR-based mutagenesis of *pUASTattB-Axin-V5* [37]. The resulting *AxinΔRGS-V5* fragment was digested with *KpnI* and *XbaI*, and then inserted into the *pUASTattB* vector at the *KpnI* and *XbaI* sites. Transgenic flies were generated using site-specific integration at the *attP33* site using phiC31-based integration [67].

Transgenes *pUASTattB-AxinΔArm-V5*, *pUASTattB-AxinΔPP2-V5* and *pUASTattB-AxinΔDIX-V5* were generated similarly, with deleted residues indicated in S1 Table.

Other stocks: *UASTattB-Axin-V5* at the *attP33* and *attP40* sites [37], *UASTattB-AxinΔTBD* [37], *C765-Gal4* (Bloomington Drosophila Stock Center, BDSC), *Apc1*^*Q8*^ [68] and *Apc2*^*19*.*3*^ [44]. The maternal *α4-Gal4*:*VP16* driver (*mat-Gal4*; line 67) contains the maternal *tubulin* promoter from *αTub67C* and the 3' UTR from *αTub84B* [47,69]. All crosses were performed at 25°C.

### Antibodies

The primary antibodies used for immunostaining were mouse anti-V5 (1:5000; Invitrogen), mouse anti-Wingless (1:200, 4D4 concentrated antibody, Developmental Studies Hybridoma Bank, DSHB). The secondary antibodies used for immunostaining were goat or donkey Alexa Fluor 488 or 555 conjugates (1:400; Invitrogen). The primary antibodies used for immunoblotting were mouse anti-V5 (1:5000, Invitrogen), guinea pig anti-Axin (1:1000, [54]), rabbit anti-Kinesin Heavy Chain (1:10000, Cytoskeleton), mouse anti-Arm (1:100, N2 7A1, DSHB), mouse anti-alpha-Tubulin (1:10000, DM1A, Sigma), rabbit anti-alpha-Tubulin (1:10000, Sigma), rabbit anti-Gluthathione-S-Transferase (1:10000, Invitrogen), guinea pig anti-Apc2 (GP10) 1:5000 [44]; rabbit anti-phospho-LRP6 [Thr1572] (1:1000, Millipore), rat anti-Dishevelled (1:1000, [70]), and guinea pig anti-Arrow (1:1000, [71]). The secondary antibodies used for immunoblotting were: goat anti-rabbit HRP conjugate (1:10000, Biorad), goat anti-mouse HRP conjugate (1:10000, Biorad), and goat anti-guinea pig HRP conjugate (1:10000, Jackson ImmunoResearch).

### Immunostaining and immunoblotting

For immunostaining, embryos were fixed in 4% formaldehyde, and rehydrated in PBT (phosphate buffered saline [PBS], 0.1% Tween-20, and 1% bovine serum albumin [BSA]). Following incubation for one hour in blocking solution (PBS, 0.1% Tween-20, 10% BSA), embryos were incubated overnight at 4°C with primary antibodies in PBT. After washing with PTw (PBS, 0.1% Tween-20), embryos were incubated with secondary antibodies for one hour at room temperature. Embryos were then washed with PTw and mounted in Prolong Gold (Invitrogen). Fluorescent images were obtained on a Nikon NIS confocal microscope and a Zeiss Axioskop 2 plus fluorescence microscope, and processed and assembled using Adobe Photoshop CS5 and Adobe Illustrator CS5.

For immunoblots, third instar larvae were dissected in cold PBS to remove salivary glands, fat body, gut, and carcass. After removal of PBS, 4X Laemmli loading buffer supplemented with 1M DTT was added and the lysates were vortexed briefly. For embryonic lysates, embryos were lysed in lysis buffer (50mM Tris-HCl [pH 8.0], 100 mM NaCl, 1% NP-40, 10% glycerol, 1.5 mM EDTA [pH 8.0]), supplemented with phosphatase and protease inhibitor cocktail (1:100, Thermo Scientific) and 1μM of the poly(ADP-ribose) glycohydrolase inhibitor ADP-HPD (Enzo Life Sciences). For S2R+ cell lysates used in immunoblots, cells were washed with cold PBS and lysed in 4X Laemmli buffer supplemented with 1M DTT. All the lysates were incubated for 5 minutes at 100°C before SDS-PAGE analysis. Quantification of immunoblots was performed with ImageJ (Wayne Rasband, National Institutes of Health).

### WWE pull-down assay

For WWE pull downs (pd), GST-WWE beads were generated as described previously [49]. S2R+ cells were treated as indicated, then washed once with cold 1X PBS and lysed in RIPA buffer (50mM Tris [pH 8.5], 300 mM NaCl, 1% NP-40, 0.5% sodium deoxycholate, and 0.1% SDS) supplemented with 1uM ADP-HPD and protease and phosphatase inhibitor cocktail (1:100). Lysates were incubated with GST-WWE beads overnight at 4°C. Following incubation, beads were washed four times in wash buffer (50mM Tris-HCl [pH 8.0], 150mM NaCl, 1% NP-40, 10% glycerol, 1.5mM EDTA [pH 8.0]) supplemented with 1uM ADP-HPD and protease and phosphatase inhibitor cocktail (1:100). Bound materials were eluted with 4X sample buffer and resolved by SDS-PAGE, transferred to nitrocellulose membranes and blotted with the indicated antibodies.

### Cell culture and transfection

S2R+ cells and S2TubWg cells were obtained from the Drosophila Genomics Resource Center. Cells were maintained a 25°C in Schneider’s Drosophila medium + L-glutamine (Gibco) supplemented with 10% (V/V) fetal bovine serum (FBS, Gibco) and 0.1 mg/mL penicillin/streptomycin (Invitrogen) (Complete medium). Cells were transiently transfected using calcium-phosphate DNA precipitation [72].

Plasmids used for transfection of Drosophila S2R+ cells were *pAc5*.*1-Axin-V5* and *pAc5*.*1-AxinΔRGS-V5*. To generate the *pAc5*.*1-AxinΔRGS-V5* plasmid, a fragment encoding *AxinΔRGS-V5* from *pUASTattB-AxinΔRGS-V5* was digested using *KpnI* and *XbaI*. The resulting fragment was inserted into the *pAc5*.*1* vector (Invitrogen) at the *KpnI* and *XbaI* sites.

### Wingless conditioned medium

To collect Wingless conditioned medium (Wg CM), S2TubWg cells (Drosophila Genomics Resource Center) were grown to confluence, then split 1:3 and incubated at 25°C for 72 hours. Cells were then resuspended in the media and centrifuged at 1000 x rpm for 5 minutes at room temperature; the supernatant was centrifuged again at 5000 x rpm for 5 minutes at room temperature. The resulting supernatant contained the Wingless conditioned medium, which was stored at 4°C. To treat cells with Wg CM, cells were washed 1X with serum-free, antibiotic-free Schneider’s medium; Wg CM or complete medium (CTR) was added and cells were incubated at 25°C for 1 hour.

### dsRNA generation and RNAi-mediated knockdown

The generation of double-stranded RNAs (dsRNAs) and dsRNA-mediated knockdown were performed as described previously [73]. Briefly, DNA templates of 200–900 nucleotides in length targeting *dsh*, *CK1α*, *GSK3*, *Apc1*, *Apc2* and *white* (negative control) were generated by PCR from genomic DNA extracted from S2R+ cells. PCR templates contained T7 promoter sequences on both ends. The DNA templates were amplified using the following primer pairs:

*white*: forward 5’-T7- ACCTGTGGACGCCAAGG-3’ and reverse 5’-T7- AAAAGAAGTCGACGGCTTC-3’ (sequence from [49]).

*dishevelled 39144*: forward 5’*-*T7*-* TCTGGTGAAGATCCCCATTC-3’ and reverse 5’-T7-CATGCCCAATTCACACTCAC -3’ (sequence from Drosophila RNAi Screening Center).

*dishevelled 19803*: forward 5’-T7- GCGCCCAGCATGTCG and reverse 5’-T7- AACGATCTCCTCGAGGTTA-3’ (sequence from Drosophila RNAi Screening Center).

*CK1α*: forward 5’-T7- CACCCTGGTCATGGACC-3’ and reverse 5-T7- TCGAAGCGCAGGCTACG-3’ (sequence from [74]).

*GSK3 23946*: forward 5’*-*T7*-* AGCTACGCATGGAGGGTAA-3’ and reverse 5’-T7-TTACCAGATCCGGGTCCAC-3’ (sequence from Drosophila RNAi Screening Center).

*GSK3 40670*: forward 5’*-*T7*-* CGAGCCGAATGTATCGTAT-3’ and reverse 5’-T7-TTCTGCCATGGATGACTCTT-3’ (sequence from Drosophila RNAi Screening Center).

*Apc1*: forward 5’-T7-ACCATTCGTAGCTACTGCACCGAA-3’ and reverse 5’-T7-ATTGATGGCTATTGGCTGCGAGGA-3’.

*Apc2*: forward 5’-T7- GTCCACAATAATCCGGA-3’ and reverse 5’-T7-GTATTGCTGGTCCTCGGGACA-3’.

dsRNAs were transcribed from PCR generated templates using the T7 Megascript kit (Ambion) according to manufacturer’s instructions. For RNAi-mediated knockdown, S2R+ cells were plated in 10 cm^2^ plates with 2.5 mL of serum-free, antibiotic-free Schneider’s medium + L-glutamine. 25 μg of each dsRNA was added to the medium and cells were incubated with gentle rotation at room temperature for 1 hour. Following incubation, 2.5 mL of complete medium were added and cells were incubated at 25°C. After 24 hours, medium was removed from the cells. This procedure was repeated once every 24 hours for a total of 96 hours. For GSK3 knockdown, an equivalent amount of *GSK3 23946* and *GSK3 23946* dsRNA (25 μg of each) were mixed and added to the medium, 50 μg of *white* or *CK1* dsRNA were used in the same experiments.

### Phosphatase treatment

S2R+ cells or third instar larvae were lysed in lysis buffer (1% NP-40, 150 mM NaCl, 50mM Tris-HCl, 50 mM NaF) supplemented with protease inhibitor cocktail (Protease Arrest, GBiosciences). Lysates were treated with λ protein phosphatase for 30 minutes according to manufacturer’s instructions (NEB).

### Immunoprecipitation

For immunoprecipitation experiments, S2R+ cells were harvested 48 hours after transfection, washed with 1X PBS, then lysed in lysis buffer (50mM Tris-HCl [pH 8.0], 100mM NaCl, 1% NP-40, 10% glycerol, 1.5mM EDTA [pH 8.0]) supplemented with 1uM ADP-HPD (Enzo Life Sciences) and phosphatase and protease inhibitor cocktail (1:100, Thermo Scientific). Lysates were incubated with mouse anti-V5 antibody (Invitrogen) overnight at 4°C, followed by addition of protein A/G-sepharose beads (Santa Cruz) for 1 hour at 4°C. Beads were washed three times with wash buffer (50mM Tris-HCl [pH 8.0], 150mM NaCl, 1% NP-40, 10% glycerol, 1.5mM EDTA [pH 8.0]) supplemented with 1uM ADP-HPD and phosphatase and protease inhibitor cocktail (1:100), and boiled with 4X sample buffer supplemented with 1M DTT. Samples were resolved by SDS-PAGE and immunoblotted with the indicated antibodies.

### Study design and statistical analysis

Student’s t-test with Welch’s correction was performed using Prism (GraphPad Software Inc., CA, USA) to compare two groups for all data sets. P values are provided in the figure legends.

## Supporting information

S1 Fig An *in vivo* system to analyze Axin regulation by Wnt signaling near physiological levels. Confocal images of embryos expressing *Axin-V5* driven by *mat-Gal4* driver stained with V5 and Wg antibodies. (A-C) Axin is uniformly distributed in the ectoderm at the onset of Wg expression. At this stage, the initial expression of Wg in segmental stripes is weak (B). (D-F) By 120 minutes after the onset of Wg expression, Axin levels decrease near the position of Wg stripes (asterisks).
(TIF)

S2 Fig Analysis of the degradation of various Axin mutants induced by Wg exposure. Stage 10 embryos expressing the indicated transgenes driven by the *mat-Gal4* driver were stained with V5 and Wg antibodies. The levels of various Axin mutants are decreased in cells responding to Wg (asterisks), suggesting the Tankyrase (A-C), Apc (D-F), Armadillo (G-I), PP2 (J-L) and Dishevelled-binding domains (M-O) are dispensable for Wg-dependent Axin proteolysis.
(TIF)

S3 Fig Apc is dispensable for Axin degradation induced by Wg exposure. (A-F) Immunostaining of stage 10 wild-type embryos expressing *Axin-V5* driven by the *mat-Gal4* driver with V5 and Wg antibodies. By 120 minutes after the onset of Wg expression, Axin levels are decreased in cells responding to Wg (asterisks). High magnification images are shown in (D-F). (G-L) Stage 10 embryos in which *Apc2* is completely inactivated maternally and zygotically and *Apc1* is reduced zygotically. Embryos in which *Axin-V5* is driven by the *mat-Gal4* driver were stained with V5 and Wg antibodies. Similar to wild-type embryos, by 120 minutes after the onset of Wg expression, Axin levels are decreased in cells responding to Wg (asterisks). High magnification images are shown in (J-L).
(TIF)

S4 Fig Expression of Axin-V5 at higher levels does not affect Wg-induced Axin degradation. (A-C) Immunostaining of stage 10 embryos expressing *attP40 Axin-V5* driven by the *mat-Gal4* driver with V5 and Wg antibodies. By 120 minutes after onset of Wg exposure, Axin-V5 staining is decreased in cells responding to Wg (asterisks).
(TIF)

S5 Fig Detection of Axin-V5 phosphorylation *in vivo*. Lysates from third instar larvae expressing *Axin-V5* with the *C765-Gal4* driver were treated with λ protein phosphatase and analyzed by immunoblotting with V5 antibody. Axin-V5 is phosphorylated when expressed in third instar larvae. Kinesin was used as a loading control.
(TIF)

S1 Table Schematic of Drosophila Axin deletions. (PDF)
