# Supplementary figures and images for: Axin phosphorylation in both Wnt-off and Wnt-on states requires the tumor suppressor APC
Source: PLoS Genet. 2018 Feb 6;14(2):e1007178. doi: 10.1371/journal.pgen.1007178 (PMC5800574; doi:10.1371/journal.pgen.1007178)

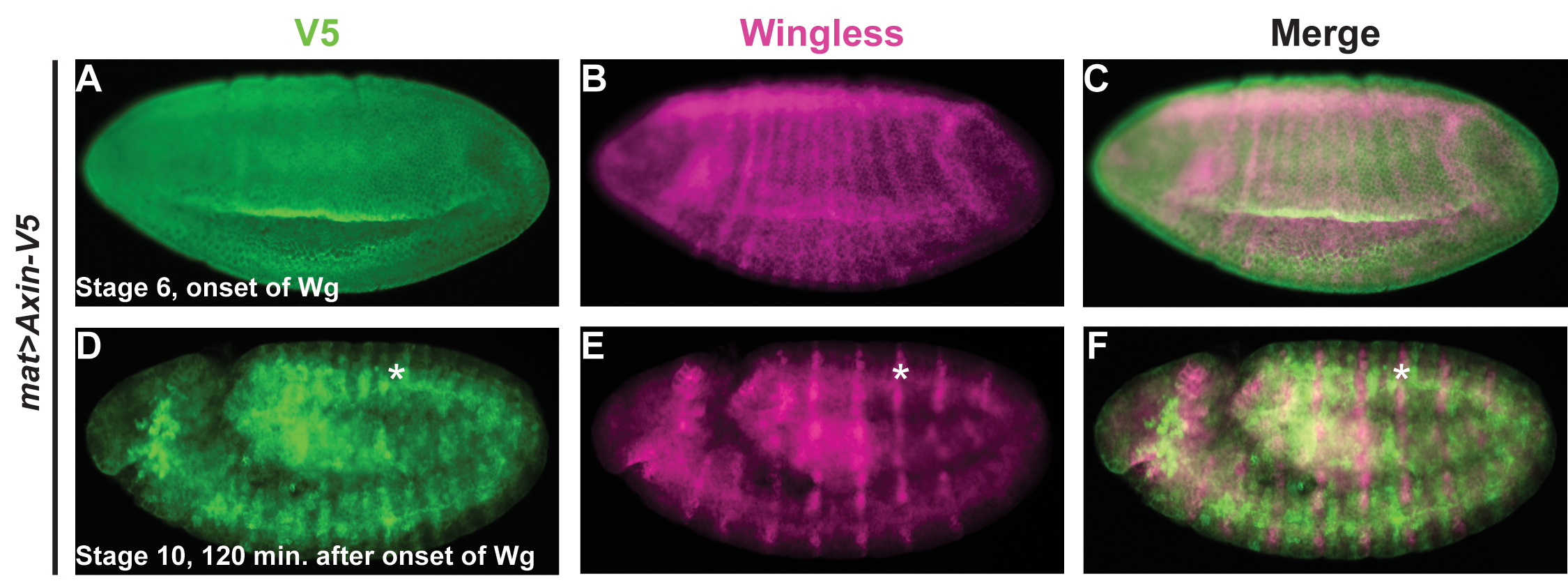

Supplement: S1 Fig — Confocal images of embryos expressing Axin-V5 driven by mat-Gal4 driver stained with V5 and Wg antibodies. (A-C) Axin is uniformly distributed in the ectoderm at the onset of Wg expression. At this stage, the initial expression of Wg in segmental stripes is weak (B). (D-F) By 120 minutes after the onset of Wg expression, Axin levels decrease near the position of Wg stripes (asterisks). (TIF) [file pgen.1007178.s001.tif]

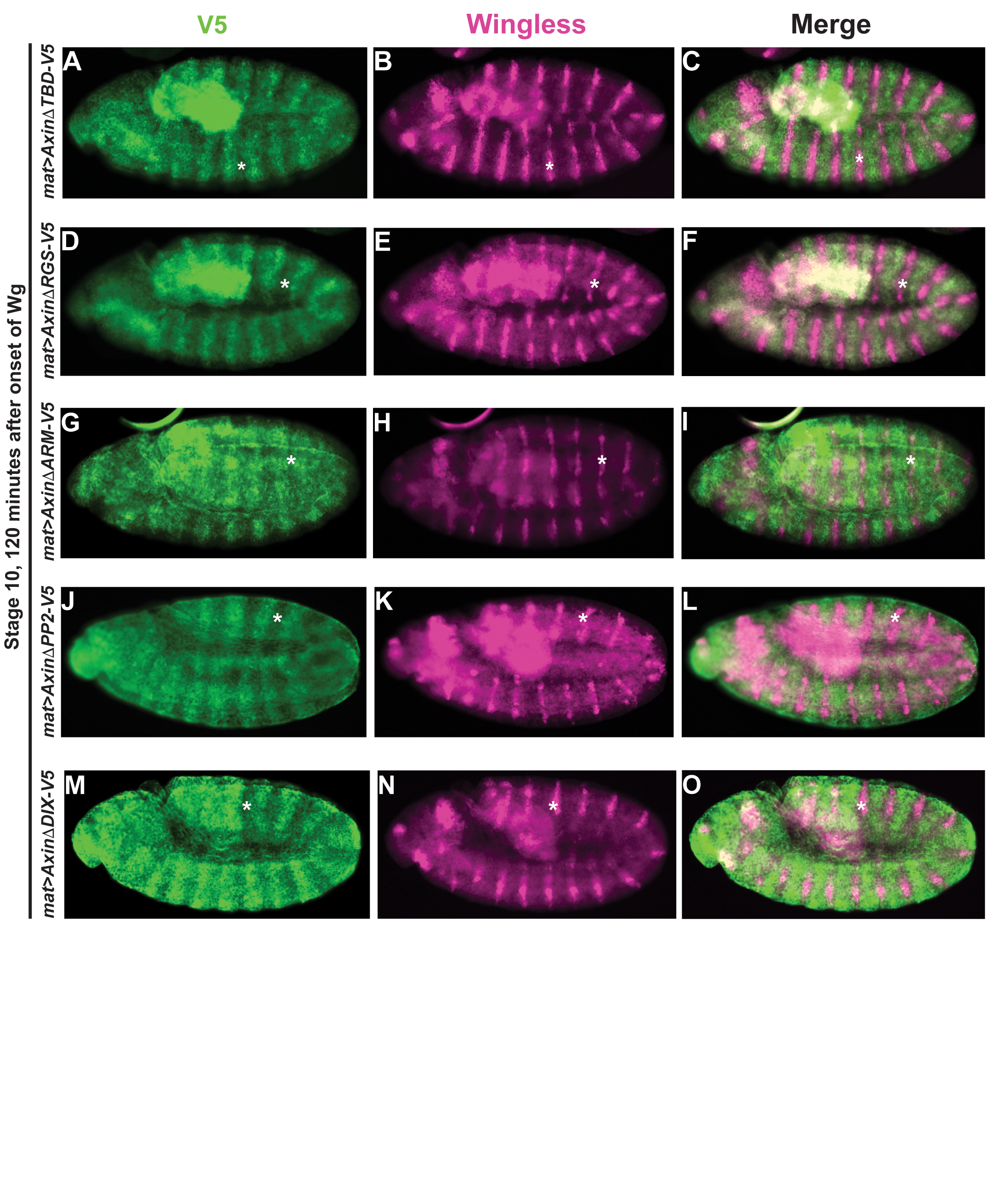

Supplement: S2 Fig — Stage 10 embryos expressing the indicated transgenes driven by the mat-Gal4 driver were stained with V5 and Wg antibodies. The levels of various Axin mutants are decreased in cells responding to Wg (asterisks), suggesting the Tankyrase (A-C), Apc (D-F), Armadillo (G-I), PP2 (J-L) and Dishevelled-binding domains (M-O) are dispensable for Wg-dependent Axin proteolysis. (TIF) [file pgen.1007178.s002.tif]

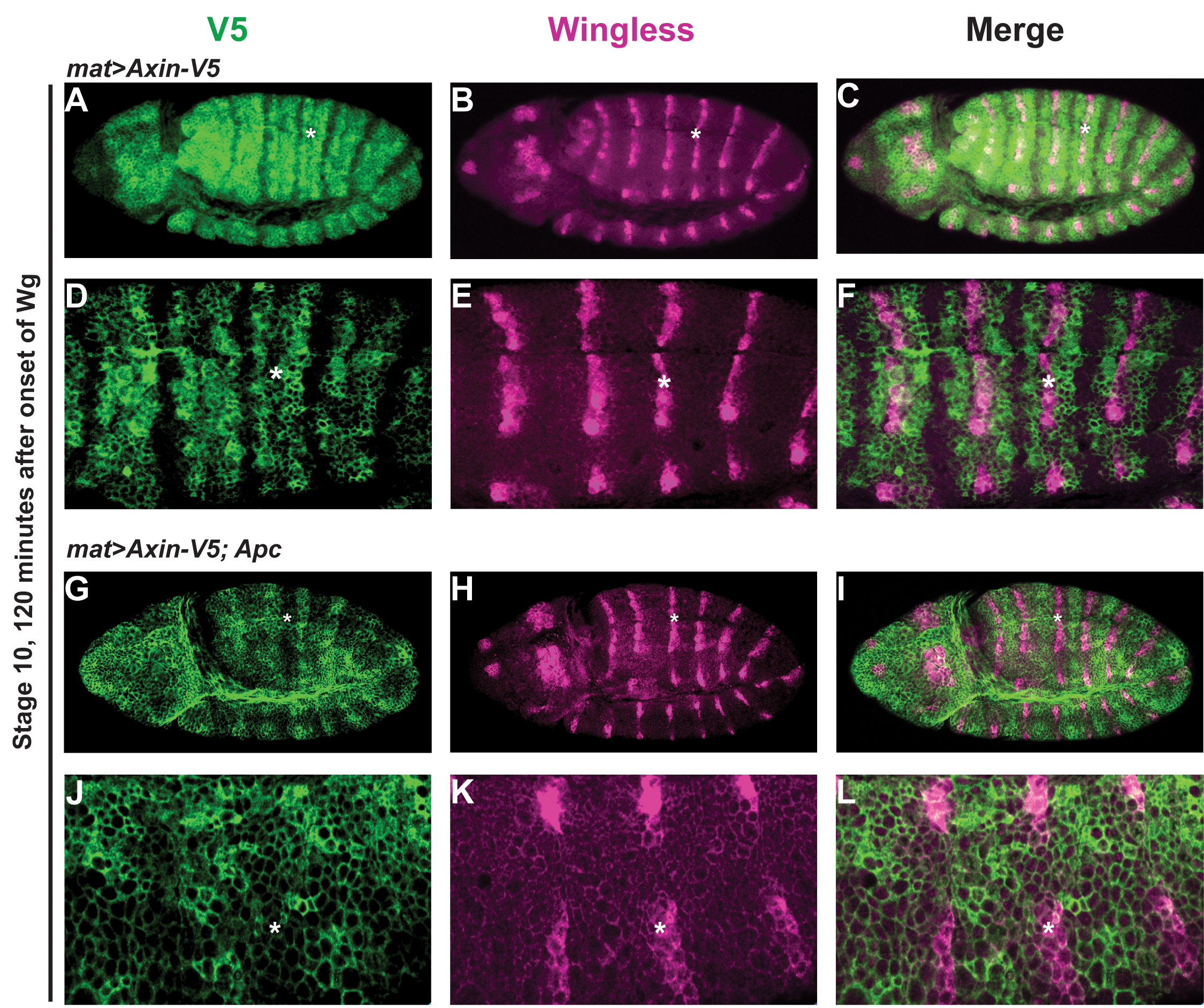

Supplement: S3 Fig — (A-F) Immunostaining of stage 10 wild-type embryos expressing Axin-V5 driven by the mat-Gal4 driver with V5 and Wg antibodies. By 120 minutes after the onset of Wg expression, Axin levels are decreased in cells responding to Wg (asterisks). High magnification images are shown in (D-F). (G-L) Stage 10 embryos in which Apc2 is completely inactivated maternally and zygotically and Apc1 is reduced zygotically. Embryos in which Axin-V5 is driven by the mat-Gal4 driver were stained with V5 and Wg antibodies. Similar to wild-type embryos, by 120 minutes after the onset of Wg expression, Axin levels are decreased in cells responding to Wg (asterisks). High magnification images are shown in (J-L). (TIF) [file pgen.1007178.s003.tif]

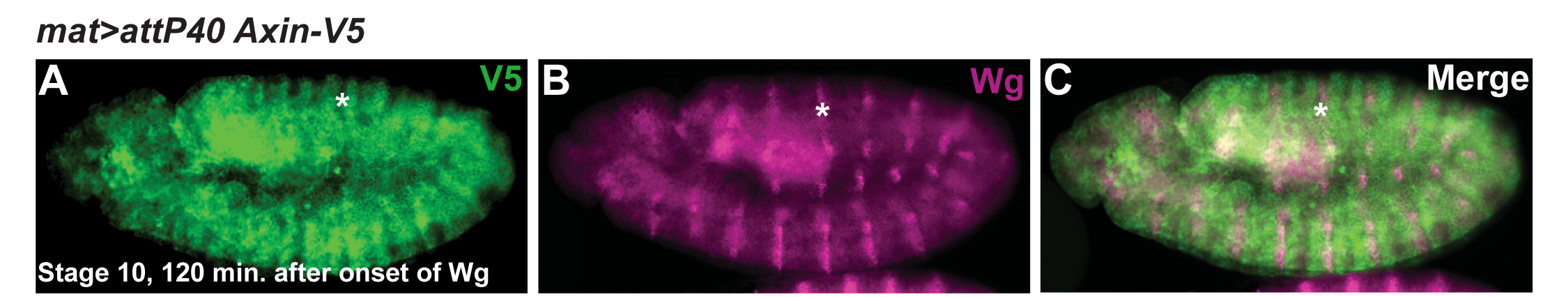

Supplement: S4 Fig — (A-C) Immunostaining of stage 10 embryos expressing attP40 Axin-V5 driven by the mat-Gal4 driver with V5 and Wg antibodies. By 120 minutes after onset of Wg exposure, Axin-V5 staining is decreased in cells responding to Wg (asterisks). (TIF) [file pgen.1007178.s004.tif]

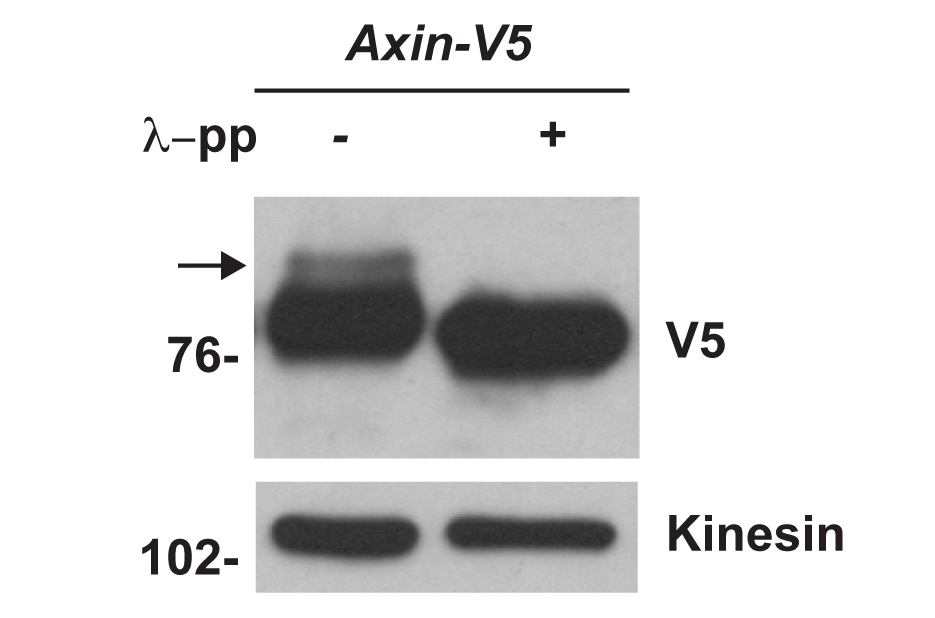

Supplement: S5 Fig — Lysates from third instar larvae expressing Axin-V5 with the C765-Gal4 driver were treated with λ protein phosphatase and analyzed by immunoblotting with V5 antibody. Axin-V5 is phosphorylated when expressed in third instar larvae. Kinesin was used as a loading control. (TIF) [file pgen.1007178.s005.tif]
