# Supplementary material for: Axin phosphorylation in both Wnt-off and Wnt-on states requires the tumor suppressor APC
Source: PLoS Genet. 2018 Feb 6;14(2):e1007178. doi: 10.1371/journal.pgen.1007178 (PMC5800574; doi:10.1371/journal.pgen.1007178)
Supplement: S1 Table — (PDF) [file pgen.1007178.s006.pdf]

	Schematic of Drosophila Axin and mutants	Early stripes	ADP-ribosylated
Axin-V5	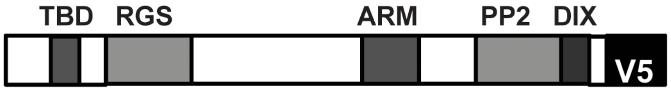	Yes	Yes
AxinΔTBD-V5	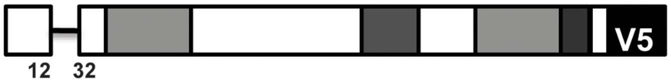	No	No
AxinΔRGS-V5	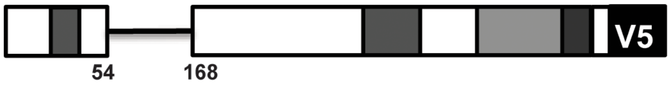	No	Yes
AxinΔARM-V5	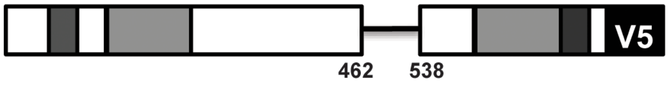	Yes	Yes
AxinΔPP2-V5	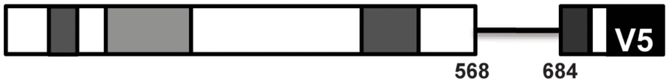	No	No
AxinΔDIX-V5	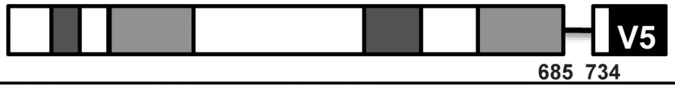	No	No
